# Oral health knowledge, attitudes, and practices of people living with mental illness: a mixed-methods systematic review

**DOI:** 10.1186/s12889-024-19713-1

**Published:** 2024-08-21

**Authors:** Alisha Maree Johnson, Amanda Kenny, Lucie Ramjan, Toby Raeburn, Ajesh George

**Affiliations:** 1 School of Nursing and Midwifery, Western Sydney University, Penrith, NSW 2751 Australia; 2 South Western Sydney Local Health District Mental Health Service, Liverpool, NSW Australia; 3 Australian Centre for Integration of Oral Health (ACIOH), Ingham Institute for Applied Medical, Research, Liverpool, NSW 1871 Australia; 4 Faculty of Medicine and Health, School of Dentistry, The University of Sydney, Camperdown, NSW 2006 Australia; 5 School of Nursing, Midwifery and Paramedicine, Australian Catholic University, North Sydney, NSW 2060 Australia; 6 La Trobe Rural Health School, La Trobe University, Bendigo, VIC 3550 Australia; 7 University of Lincoln, Lincoln, LN6 7TS United Kingdom; 8 School of Nursing, University of Wollongong, Wollongong, NSW 2522 Australia

**Keywords:** Mental health, Mental disorder, Oral health, Knowledge, Practice

## Abstract

People living with mental illness experience poorer oral health outcomes compared to the general population, yet little is known about their oral health knowledge, attitudes, and practices. The aim of this mixed-methods systematic review was to synthesise evidence regarding oral health knowledge, attitudes, and practices of people living with mental illness to inform preventative strategies and interventions. Database searches were conducted in PubMed, MEDLINE, PsycINFO, CINAHL, ProQuest, and Scopus with no limitations placed on the year of study. All studies available in the English language, that explored the oral health knowledge, attitudes, and/or practices of people with a mental illness were included. Articles were excluded if they primarily pertained to intellectual disability, behavioural and psychological symptoms of dementia, drug and alcohol or substance use, or eating disorders. A thematic synthesis was undertaken of 36 studies (26 high-moderate quality), resulting in 3 themes and 9 sub-themes. Study participants ranged from *n* = 7 to *n* = 1095 and aged between 15–83 years with most having a diagnosis of schizophrenia, schizoaffective, or bipolar affective disorder. People diagnosed with a mental illness were found to have limited oral health knowledge, particularly regarding the effects of psychotropic medication. Various barriers to oral health care were identified, including high dental costs, the negative impact of mental illness, dental fears, lack of priority, and poor communication with dental and health care providers. Study participants often displayed a reduced frequency of tooth brushing and dental visits. The findings highlight the potential for mental health care providers, oral health and dental professionals, mental health consumers, and carers to work together more closely to improve oral health outcomes for people with mental illness. The systematic review protocol is registered with the International Prospective Register of Systematic Reviews (PROSPERO), (registration ID CRD42022352122).

**Supplementary Information:**

The online version contains supplementary material available at 10.1186/s12889-024-19713-1.

## Introduction

Mental illness encompasses a broad range of conditions that affects a person’s way of thinking or behaviour causing distress or impairment [1]. Globally, the impact of mental illness is significant and is ranked in the top 20 causes of disease burden [2]. It is estimated that approximately 1 in 8 people worldwide currently live with a diagnosable mental illness [1].

The impacts of mental illness are multifaceted, with people experiencing social, economic, and physical health adversities [3, 4]. Poor overall health, reduced life expectancy [5, 6], and increased risk of metabolic-related illnesses secondary to psychotropic medication use and illness symptomology [7] are often reported. Amotivation and anhedonia (loss of ability to feel pleasure) results in reduced engagement in self-care activities, including self-hygiene and exercise [8]. This may contribute to a sedentary lifestyle, poor diet with reduced fruit and vegetable intake [9, 10] and increased intake of sugary drinks [11]. Obesity secondary to antipsychotic medication use [12] can occur, and higher rates of smoking are reported [13].

People living with mental illness experience significantly poorer oral health outcomes compared to the general population [14–19]. They are at higher risk of tooth loss and oral health diseases including periodontal disease and dental caries [18, 20]. Psychotropic medications, used in treatment are associated with hyposalivation, causing dry mouth and resultant dental caries [20, 21]. However, authors Persson, Olin and Ostman [22] state that this population may have a poor understanding of the impact of mental illness on oral health which is secondary to feelings of shame regarding their oral health status. They may have had previous traumatic experiences that influenced decisions about seeking dental care [22]. Reduced self-care such as brushing teeth can impact a person’s oral health status [23]. For many people who experience mental illness, the financial cost of accessing regular dental care may be prohibitively expensive [24]. The focus of most systematic reviews has been on highlighting the oral health adversities for this population. To date, no review has focused on the oral health knowledge, attitudes, and practices of people living with mental illness. This review addresses this gap by answering the question what are the oral health knowledge, attitudes, and practices of people living with mental illness?

## Aims

The aim of this systematic review was to synthesise the available evidence regarding oral health knowledge, attitudes, and practices of people living with a mental illness to help inform future preventative strategies and interventions.

## Definition of terms

The term ‘people living with a mental illness’ has been used throughout this review to include any person who has received a clinical diagnosis of a mental illness or mental disorder.

Knowledge includes understanding the relationship between oral health and mental illness, complications and impact of prescribed medication, knowledge on seeking out oral health resources and causes of poor oral health.

Attitudes refers to a person’s perception towards oral health, including perceived barriers to attending to oral health and attending dental visits.

Practices included the actions that a person engages in to maintain oral health, including tooth brushing frequency, type of tooth brushing aid used to brush teeth and dental visits. For the purpose of this review, practices did not include dietary practices.

## Methods

This mixed-methods review was guided by the work of Khan et al [25] and included developing the review question, identifying relevant studies, quality assessment, and summarising and interpreting the evidence. The Preferred Reporting Items for Systematic Reviews and Meta-Analyses (PRISMA) statement and reporting checklist guided reporting [26]. The systematic review protocol is registered with the International Prospective Register of Systematic Reviews (PROSPERO), (registration ID CRD42022352122).

### Eligibility criteria

Studies that met the following criteria were included: published in the English language, participants had a diagnosis of mental illness, and the study explored at least one study outcome (knowledge, attitude, or practice toward oral health). All studies including qualitative, quantitative, and mixed methods studies were eligible for inclusion to ensure the breadth of evidence was captured. Any experimental studies with a pre-survey component were also included. No restrictions were placed on the year of publication, quality, or study setting to ensure all available literature was included. Studies of people with primary alcohol or substance use disorders, intellectual disability, and behavioural and psychological symptoms of dementia were excluded due to the additional oral health complexities associated with these co-morbidities. Studies that focused on people who experience eating disorders were also excluded due to the additional oral health complications that arise secondary to an eating disorder.

### Data sources, search strategy, and study selection

A systematic search of all peer-reviewed literature published up until August 2022 was undertaken in consultation with a university librarian. Six databases were searched (PubMed, MEDLINE, PsychINFO, CINAHL, ProQuest, and Scopus) using various search strategies (see supplementary file for an example). Keywords used in the search included: Mental illness*, mental disorder, psychiatry*, oral health, oral health care, oral hygiene, dental care, knowledge, attitude*, practice*, perception, and awareness. Database-specific index terms were used in the search and reference lists of included studies were hand-searched. Combination search terms including ‘Boolean’ operators were used. Within each database search, an English language filter was also applied. A repeat database search was conducted in October 2023 to ensure all studies were captured.

**Fig. 1:**
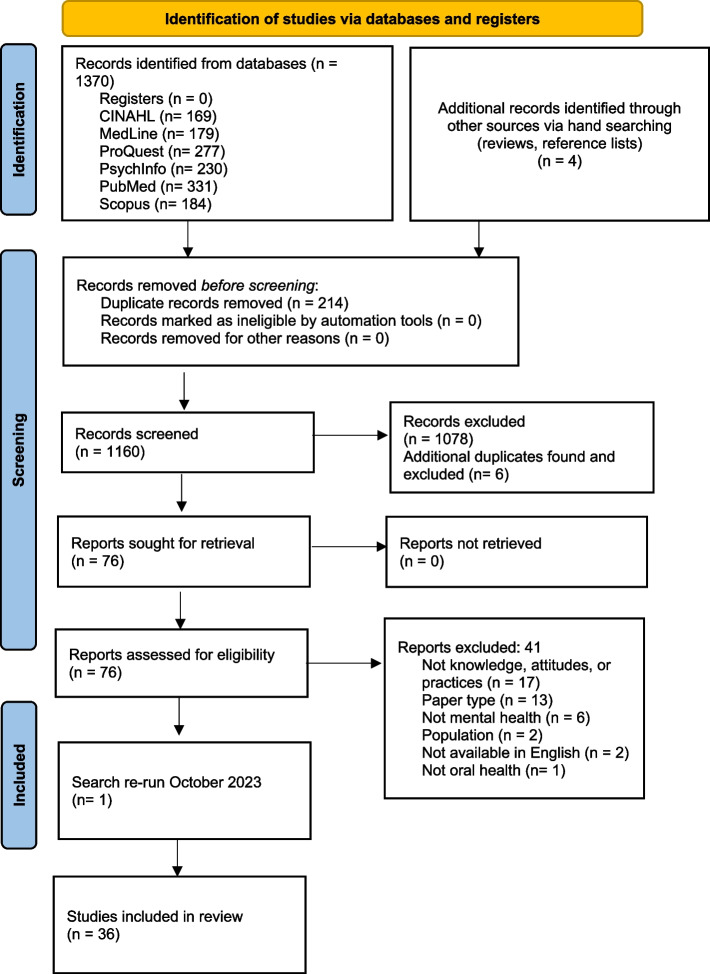
PRISMA flow chart of the study selection process

The results from database searches were imported from Endnote bibliographic software into the Cochrane systematic review management program, Covidence, where duplicate references were removed. Covidence, was used to manage the screening process. Titles and abstracts of studies were assessed by two separate investigators (AJ: all studies and AG: 67%, AK: 17%, TR: 1%, LR: 15%) using the inclusion and exclusion criteria. The full text was screened by two separate investigators (AJ and AG). Any discrepancies between reviewers were resolved with team discussion. The selection process and the final studies included in the systematic review are illustrated in Fig. 1 using the current PRISMA flowchart [26].

### Data extraction and data synthesis

Data extraction tables were developed by the team to extract relevant information from the included studies. The information included author, year of publication, country, article type, study characteristics (design, participant demographics), study findings, and quality assessment rating.

A thematic synthesis approach was used to collate, analyse, and present the findings of the study [27]. The full texts of all included studies were closely read and re-read, and codes were generated using a hybrid inductive and deductive approach. Codes were grouped into meaningful themes and subthemes that reflected the overall study aim. The generated themes were reviewed by a second investigator (AG) and revised accordingly. A team meeting was held to explore interpretations and finalise themes. Direct quotes are used to support the themes generated. As statistic pooling was not possible due to a lack of homogeneity within the included studies, quantitative data are presented in narrative format to support the themes and sub-themes. Three major themes and nine subthemes were identified from the studies- see Table 1.

**Table 1:** Themes and subthemes along with contributing data

Theme and subthemes	Type of data contributing to the theme
**Oral health knowledge**	- Randomised control trial (*n* = 2) [28, 29]
Preventative oral health care	- Cross-sectional (*n* = 2) [30, 31]
Side effects of psychotropic medication	- Cohort (*n* = 2) [32, 33]
	- Qualitative (*n* = 1) [24]
**Oral health attitudes**	- Randomised control trial (*n* = 2) [29, 34]
Impact of mental illness	- Cross-sectional (*n* = 4) [35–38]
Oral health not a priority	- Qualitative (*n* = 7) [22, 39–44]
**Oral health practices**	- Randomised controlled trial (*n* = 2) [29, 34]
Tooth brushing aids	- Cross-sectional (*n* = 19) [30, 31, 35–38, 45–57]
Frequency of brushing	
Dental visits	
Cost	
Poor communication with dentists and health care providers	
Practical barriers	- Case–control (*n* = 2) [58, 59]
Dental fears	- Cohort (*n* = 4) [32, 33, 60, 61]
	- Qualitative (*n* = 6) [24, 39–42, 44]

## Quality assessment

The quality of studies was assessed using the Joanna Briggs Institute (JBI) Checklist that aligned with the methodology of corresponding studies [62] and included the checklist for qualitative research [63], analytical cross sectional studies [64], cohort studies [65], randomized controlled trials [66], and case control studies [67]. Two separate investigators (AJ: all studies and LR: 50%/TR: 50%) scored the included studies by assigning 1 point for each applicable item. A third author (AG) was consulted to resolve any discrepancies. After ascertaining consensus among authors, cut off values were established including, 0–59% considered poor quality, 60–79% considered moderate quality, and 80% or greater considered high quality [68]. No articles were excluded in this systematic review based on quality appraisal.

## Results

### Insert PRISMA

There were 36 studies included in this review, published between 1995 and 2022 and conducted in 15 countries including, United Kingdom (6/36), USA, (5/36), India (4/35), Denmark (4/36), Australia (2/36), Netherlands (2/36), Taiwan (2/36), Japan (2/36), Turkey (2/36), Sweden (2/36), France (1/36), Norway (1/36), Ethiopia (1/36), Brazil (1/36), and Singapore (1/36). The sample size ranged from *n* = 7 [39] to  21,417 [60] . The age of participants ranged from 15 to 83 years and consisted of mostly male participants.

Most authors (32/36) reported on a diagnosis of the study population with 23 of the 32 studies having a combination of disorders within their sample. The remaining studies had populations with schizophrenia (*n* = 6), first episode psychosis (*n* = 2), and psychotic illness in general (*n* = 1). Four studies did not include a diagnosis of the participants (See Table 2). Of the 36 studies included in this review, the majority were conducted in a community setting, with 10 conducted in an in-patient (hospital) setting. Of the 36 studies, 27 were quantitative and eight were qualitative, and one was mixed methods, with two of the 36 included studies using a validated tool. The quality of studies was assessed with 21 studies considered high, five considered moderate, and 10 considered poor (See Tables 3 and 4). Studies rated as having poor quality were lacking across certain assessment criteria, the most common being lack of identification of confounding variables and lack of measurement of exposure/outcomes in a valid and reliable way (see Table 4). In seven of the 10 studies some of the assessment criteria were also ‘unclear’ which resulted in a lower quality score. There were no significant discrepancies though between quality of studies and their findings. The themes that emerged from the poor quality studies followed the same trends as the moderate and high quality studies.

**Table 2:** Study demographics

Author, year of publication	Country	Methodology/ Method	Sample size/ Setting	Sample characteristics		
				Age	Gender	Diagnosis and percentage of participants
Adams et al., 2018 [34]	UK	Quantitative/ randomised controlled trial	*n* = 1095 Community	18 and over	M = 66.3% (726), F = 33.7% (369)	Psychotic illness
Agarwal et al., 2021 [35]	India	Quantitative/ cross-sectional	*n* = 111 Community	15–60	M = 75% 83, F = 25%28	Schizophrenia
Alkan et al., 2015 [48]	Turkey	Quantitative/ cross-sectional	*n* = 364 Community	18–65	M = 46.4% (169), F = 53.6% (195)	Anxiety, Depression and Attention Deficit Hyperactive Disorder
Almomani et al., 2009 [28]	USA	Quantitative	*n* = 60 Community	22–62	M = 45% (27), F = 55% (33)	Schizophrenia = 57.1% Schizoaffective = 5.3% Bipolar Affective Disorder = 12.5% Depression = 32.1%
Anita et al., 2019 [49]	India	Quantitative/ cross-sectional	*n* = 304 Community	18–71	M = 52.3% (159), F = 47.7% (145)	Schizophrenia M = 36%, F = 21%, Mood disorder M = 7%, F = 46%, Organic brain damage M = 34%, F = 14%, Other M = 23%, F = 19%
Bertaud-Gounot et al., 2013 [50]	France	Quantitative/ cross-sectional	*n* = 161 In-patient	18–90	M = 59% (95), F = 41% (66)	Schizophrenia = 36.6% Mood disorders = 21.1%, Mental disorders due to psychoactive substance use = 12.4% Organic = 8.1% Mental retardation = 7.5% Neurotic, stress related and somatoform disorders = 3.7%, Disorders of psychological development = 3.7% Personality disorders = 2.5% Behavioural and emotional disorders onset in childhood and adolescents = 1.2%
Bjorkvik et al., 2021 [40]	Norway	Qualitative/ semi-structured interviews	*n* = 51 Community	22–78	M = 35% (18), F = 65% (33)	Anxiety disorders = 70.6% Mood disorders = 66.7% Psychosis in Bipolar disorder = 34.4% Severe traumatic experiences = 54.9% Cognitive disabilities = 29.4% Severe somatic illness = 47.1%
Gurbuz 2011 [55]	Istanbul, Turkey	Quantitative/ cross-sectional	*n* = 330 In-patient	22–82	M = 54.2% (179), F = 35.8% (151)	Schizophrenia = 61.8% Mental retardation = 30.6% Organic mental disorder = 3.3% Atypical psychosis = 4.2%
Hall et al., 2018 [45]	USA	Quantitative/ cross-sectional	*n* = 186 Community	18–83	M = 31.7% (59), F = 68.3% (127)	Schizophrenia = 30.7% BPAD = 23.1% Depression = 17.2% PTSD = 14.0% Anxiety = 7.5% ADHD = 4.3% Other = 1.6% prefer not to answer = 1.3%
Hede, et al., 1992 [30]	Denmark	Quantitative/ cross-sectional	*n* = 84 In-patient	20–64	M = 35.7% (30), F = 64.3% (54)	Schizophrenia = 51% Manic-depression 10% Reactive psychosis = 25% Other = 14%
Hede, B. 1995 [36]	Denmark	Quantitative/ cross-sectional	*n* = 240 Community	20–64	M = 60% (144), F = 40% (96)	Schizophrenia = 33% Mood disorders = 30% Anxiety disorders = 21% Other = 16%
Ho et al., 2018 [42]	Australia	Qualitative/ focus-groups and semi-structured interviews	*n* = 12 Community	18 and over	M = 33.3% (4), F = 66.6% (8)	Not reported
Janardhanan et al., 2011 [59]	USA	Quantitative/ cross-sectional comparison	n = 311 Community	55 +	M = 32.8% (102), F = 67.2% (209)	Schizophrenia
Kebede et al., 2012 [54]	Ethiopia	Quantitative/ cross-sectional	*n* = 240 In-patient	15–68	M = 70% (168), F = 30% (72)	Depression = 65.8% psychotic disorder = 17.5% anxiety disorder = 8.3% 2.1% = epilepsy bipolar disorder = 1.7% other = 4.6%
Khokhar, et al., 2011 [32]	UK	Quantitative/ pre-post test	*n* = 59 In-patient	22–76	M = 67.8% (40), F = 32.2% (19)	Not reported
Kuipers et al., 2018 [41]	Netherlands	Qualitative/ open-ended interviews	*n* = 30 Community	18–35	M = 60% (18), F = 40% (12)	First episode psychosis
Kuipers et al., 2022 [58]	Netherlands	Quantitative/ case–control	*n* = 81 Community	18–35	Case group: M = 52, F = ,29 Control group M = 107, F = 59	First episode psychosis
Kuo et al., 2020 [29]	Taiwan	Quantitative/ Randomised controlled trial	*n* = 58 In-patient	20 and over	NR	Schizophrenia = 87.9%, Mood disorder = 1% Organic mental illness = 4% Other = 2%
Lalloo et al., 2013 [51]	Australia	Quantitative/ cross-sectional	*n* = 50 Community	20–83	M = 80% (40), F = 20% (10)	Schizophrenia and other non-affective psychoses = 76%, Schizoaffective = 18% Bipolar Affective Disorder = 2%
Lopes et al., 2021 [52]	Brazil	Quantitative/ cross-sectional	*n* = 753 Community	NR	M = 48.6% (366), F = 51.4% (387)	Schizophrenia, depression
McKibbin et al., 2015 [24]	USA	Qualitative/semi-structured interviews	*n* = 25 Community	35–63	M = 52% (13), F = 48% (12)	Bipolar Affective Disorder = 52% Schizophrenia = 28% Schizoaffective disorder = 16% Major depression = < 1%
Mishu et al., 2022 [39]	UK	Qualitative/ interviews	*n* = 7 Community	31- > 60	M = 43% (3), F = 57% (4)	Schizophrenia = 57% Bipolar disorder = 43%
Nayak et al., 2020 [53]	India	Quantitative/ cross-sectional	Community	21–70	NR	Depression, schizophrenia, mood disorder, mania
Ngo et al., 2018 [37]	Singapore	Quantitative/ cross-sectional	*n* = 191 In-patient	24–80	M = 74.9% (143), F = 25.1% (48)	Schizophrenia = 88.5% Intellectual disability = 4.7% Other = 6.8%
Nielsen et al., 2011 [60]	Denmark	Quantitative/ prospective database study	n = 21,417 Community/ inpatient	18 and over	M = 59%, F = 41%	Schizophrenia
Persson et al., 2009 [46]	Sweden	Quantitative/ cross-sectional	*n* = 113 Community	21–64	M = 40.7% (46), F = 59.3% (67)	Schizophrenia = 33% Mood disorders = 30% Anxiety disorders = 21% Other = 16%
Persson et al., 2010 [22]	Sweden	Qualitative/ longitudinal intervention	*n* = 10 Community- supported housing	NR	M = 30% (3), F = 70% (7)	Schizophrenia
Sogi 2020 [38]	Haryana, India	Quantitative/ cross-sectional	*n* = 165 Community	18–70	M = 58.8% (97), F = 41.2% (68)	Mood disorder = 30.9% Neurotic, stress-related and somatoform disorder = 30.9 Mental and behavioural disorders due to psychoactive substance use = 22.4% Schizophrenia, schizotypal and delusional disorder = 11.5%, Epilepsy = 4.3%
Stevens et al., 2010 [33]	UK	Quantitative/ pre-post test	*N* = 65 In-patient	NR	NR	Not reported
Tani et al., 2012 [56]	Japan	Quantitative/ cross-sectional	*n* = 523 In-patient	18–87	M = 56.8% (297), F = 43.2% (226)	Schizophrenia = 97.7% Schizoaffective disorder- 0.95% Delusional disorder = 1.14% Acute and transient psychotic disorder = 0.19%
Teng et al., 2011 [31]	Taiwan	Quantitative/ cross-sectional	*n* = 200 In-patient	NR	M = 62.5% (125), F = 37.5% (75)	Schizophrenia = 61%, Bipolar Affective Disorder = 14.5% Major depressive disorder = 11.5% Organic mental disorder = 11% Other = 2%
Tredget et al., 2019 [57]	Wales	Quantitative/ cross-sectional	Community	20–82	M = 64.2%, F = 35.8%	Schizophrenia
Villadsen et al., 2017 [43]	Denmark	Qualitative/ open interviews	*n* = 23 Community	21–58	M = 47.9% (11), F = 52.1% (12)	Schizophrenia
Waplington et al., 2000 [47]	UK	Mixed/Semi-structured interviews	*n* = 70 Community	NR	M = 55.7% (39), F = 44.3% (31)	Not reported
Wright et al., 2021 [44]	USA	Qualitative/ interviews	*n* = 20 Community	21–63	M = 40% (8), F = 60% (12)	Schizophrenia, Bipolar disorder, Schizoaffective disorder, Major depressive disorder = 70% Borderline personality disorder, post-traumatic disorder, anxiety disorder, attention-deficit hyperactive disorder = 30%
Yoshii et al., 2017 [61]	Japan	Quantitative/ pre-post test	*n* = 323 Community- Day center	NR	M = 62.5% (202), F = 37.5% (121)	Schizophrenia = 64.4% Mood disorders = 23.2% other = 6.5% unknown = 5.9%

**Table 3:** Study findings

Author, Year	Findings			Quality rating
	Oral Health Knowledge	Oral Health Attitudes	Oral Health Practices	
Adams et al., 2018 [34]		**Oral health not a priority**	**Tooth brushing aids**	
		- 22% No need (to see a dentist)	- 98% owned a toothbrush	
		- 21% Not registered with a dentist	**Frequency of brushing**	
		- 17% Dental avoidance	- 50% brushed 2–3 times a day	High
			- 4% brushed between 1–2 times a day	
			- 40% brushed 1 per day-few times a week	
Agarwal et al., 2021 [35]		**Oral health not a priority**	**Tooth brushing aids**	
		- 84.7% perceived oral health as having little influence on their lives	- 72.1% aid of cleaning teeth: toothbrush	
			- 77.5% rinsed mouth after every meal	Moderate
			- 11.7% used an aid for cleaning tongue (brush or tongue cleaner)	
			**Frequency of brushing**	
			- 2.7% brushed teeth twice a day	
Alkan et al., 2015 [48]			**Frequency of brushing**	
			- 40.6% brushed once a day	
			- 29.9% twice a day	
			- 29.9% every other day	Moderate
			**Dental visits**	
			- 15.1% once a year	
			- 80.5% when had a problem	
			- 4.4% never visited dentist	
Almomani et al., 2009 [28]	- Poor oral health knowledge scores related to questions on oral health knowledge < 45% (14.7 and 15.0/34)			High
Anita et al., 2019 [49]			**Tooth brushing aids**	
			- 11% required assistance in brushing teeth and brushed teeth once a day	
			- 89% cleaned teeth with no assistance	
			**Frequency of brushing**	Moderate
			- 10% cleaned twice a day	
			- 4% were irregular in cleaning their teeth	
Bertaud-Gounot et al., 2013 [50]			**Frequency of brushing**	
			- 65% brushed once a day	
			- 18.2% brushed less than once a day	High
			- 12.6% never brushed	
Bjorkvik et al., 2021 [40]		**Impact of mental illness**- Reduced adherence to oral health care when feeling hopeless or experiencing suicidal ideation - Some participants expressed difficulty in planning dental appointments when going through bad periods or being depressed	**Barriers to accessing dental care** Practical barriers - Some participants expressed difficulties making a dental appointment - Transport and getting there on time was also identified as a barrier by some participants	Poor
Gurbuz, 2011 [55]			**Frequency of brushing** - 37.6% several times a week - 62.4% seldom/never brushed	Moderate
Hall et al., 2018 [45]			**Barriers to accessing dental care** Cost - 39% identified cost as a barrier, - 22% lack of perceived need - 8% avoiding due to anticipated problems Practical barriers - 7.4% had a fear or dislike of dentists - 2.7% transportation - 12.5% Uncertainty about coverage or difficulty accessing providers	Poor
Hede, et al., 1992 [30]	**Preventative oral health care** - 80% agreed toothbrushing mattered in dental health - 75% believed that you can improve your dental health - 55% agreed that they expect to lose most of their teeth in their lifetime **Side effects of psychotropic medications** - 86% were not aware that medication they receive may create cavities		**Frequency of brushing** - 59% claimed to brush their teeth twice a day - 24% claimed they brushed their teeth one a day - 17% claimed that they seldomly or never brushed their teeth **Dental Visit** - 66% attended the dentist regularly	Poor
Hede, B. 1995 [36]		**Oral health not a priority**- 45% of participants stated that they perceived that they needed dental treatment (due to the oral health symptoms they were experiencing)	**Frequency of brushing** Regular brushing - 42% with schizophrenia, - 75% with affective psychosis - 77% with reactive psychosis - 52% with personality disorder - 58% other Neglect of toothbrushing - 39% with schizophrenia, - 42% with affective psychosis - 39% with personality disorders - 31% with reactive psychosis - 27% with other **Dental visits** Constant regular dental visits = 31% - 70% Affective psychosis - 23% Personality disorder - 29%, Reactive psychosis - 24% other	High
Ho et al., 2018 [42]		**Impact of mental illness**- Coping with their mental health condition/dealing with life stressors made attending to preventive oral health routine challenging (managing their own lives and living with mental illness was a barrier to improving oral health competing priorities), even though they had intention to attend to OH	**Barriers to accessing dental care** Cost - Some participants expressed that they would often live with dental pain than seek treatment due to the cost associated with dental care - Participants avoided seeking a dental opinion as they feared they were unable to pay the dental bill Dental fear - Many participants expressed dental fear and anxiety as a barrier to accessing regular dental care Poor communication with dentists and health care providers - Participants that had high levels of oral health literacy, miscommunication between dental practitioner and patient still occurred - Some participants expressed that they felt that language used by dental professionals made them feel uncomfortable	Poor
Janardhanan et al., 2011 [59]			**Dental visits** - 24.2% one dental visit in previous year - 14.1% had at least two dental visits in previous year	High
Kebede et al., 2012 [54]			**Frequency of brushing** - 3.8% brushed more than once a day - 7.1% brushed once a day - 87.5% brushed irregularly - 1.7% did not brush **Tooth brushing technique** - 52.9% had incorrect brushing technique - 47.1% had correct brushing technique	Moderate
Khokhar, et al., 2011 [32]	**Preventative oral health care** 55% basic oral hygiene knowledge (brush twice/day)		**Tooth brushing aids** - 68% of participants had access to a toothbrush **Frequency of brushing** - 29% basic oral hygiene practice twice daily brushing (for those with a toothbrush) **Dental visits** - 34% were registered with a dentist - 29% had a dentist visit within one year - 50% had their dentures checked within last five years	Poor
Kuipers et al., 2018 [41]		**Oral health not a priority**- All participants expressed that they did not view dental care as a priority - Some participants identified that Dental care was not a priority when experiencing psychosis	**Barriers to accessing dental care** Cost - Current debts and finances prevented some participants going to the dentist and/or paying for health insurance to cover dental visit	High
Kuipers et al., 2022 [58]			**Tooth brushing aids** - 38.3% use of dental aid to brush **Frequency of brushing** - 49.4% low frequency brushing - 46% had short duration brushing **Dental visits** - 44.4% Low number of dental visits - 82.7% low number of dental hygienist visits	High
Kuo et al., 2020 [29]	- Poor OH knowledge scoring < 50% (4.6 and 4.3/10) on questions relating to oral health knowledge	- Moderate OH attitudes scoring average 64.6% (45.9 and 47.3 out of a range of 13–65) on questions related to attitudes towards oral health	- Poor OH practices scoring < 14% (4.1 and 3.6/10) on questions related to oral health practices including tooth brushing	Poor
Lalloo et al., 2013 [51]			**Frequency of brushing** - 52% brushed once a day - 24% twice a day **Dental visits** - 32% had not seen a dentist in past 2 years	High
Lopes, 2021 [52]			**Frequency of brushing** - 37.3% brushed less than once a day - 62.5% 2 or more times a day	High
McKibbin et al., 2015 [24]	**Preventative oral health care** - Participants expressed very basic understanding about oral health including brushing and flossing **Side effects of psychotropic medication** -Limited knowledge regarding increase risk for poor oral among adults with serious mental illness - Limited knowledge on medication side effects and impact on oral health		**Barriers to accessing dental care** Cost - Majority of participants expressed poverty as a barrier - Participants expressed difficulty in affording toothpaste and dental floss - Some participants expressed that they were unaware of what public health insurances (such as Medicaid) would cover Practical barriers - Difficulties accessing providers that accepted public health insurances, especially in rural areas	High
Mishu et al., 2022 [39]		**Impact of mental illness** - Participants expressed that mental illness, including amotivation, impacted ability to maintain oral health **Oral health not a priority** - Participants felt that oral health was not considered important from the perspective of mental health and other health professionals - Participants expressed wanting to be involved in their dental care and planning	**Barriers to accessing dental care** Cost - Participants expressed not being able to afford necessities as well as dental care Dental fear - Nature of dental treatments being intrusive Practical barriers - Difficulties accessing providers due to distance and transportation **Poor communication with dentists and health care providers** - Dentist lack of understanding about mental illness - Negative experiences, feeling discriminated against, or misunderstood prevented participants from accessing dental services	high
Nayak et al., 2020 [53]			**Frequency of brushing** - 68% brush once a day (48.8% depression, 8% schizophrenia, 6.8% mood disorder, 5.2% mania) - 31.2% brush twice or more a day (19.6% depression, 4% schizophrenia, 3.6% mood disorder, 4% mania)	Poor
Ngo et al., 2018 [37]		**Oral health not a priority**- Some participants expressed that feeling lazy was a barrier to attending oral health	**Frequency of brushing** - 48.9% brush twice a day **Barriers to accessing dental care** Practical barriers - lack of access to brushing at night (for those in wheelchairs) - long queues for brushing (limited sink numbers)	High
Nielsen et al., 2011 [60]			**Dental visits** - 43% visited the dentist within past 12 months - 31% had a regular annual dental check-up in past 3 years	High
Persson et al., 2009 [46]			**Dental visits** - 77% Regular contact with a dentist - 69% said they visited a dentist a least onc*e* a year - 15% had not been to a dentist in the past 2 years **Barriers to accessing dental care** Cost - 17% didn’t visit dentist due to financial - 43% neglected teeth due to finances Dental fear - Low anxiety reported towards dental visits (9.12 on scale between 4–20) in participants (77%) that attended dental appointments regularly	High
Persson et al., 2010 [22]		**Oral health not a priority**- Participants expressed waiting until an acute oral health situation arose then choosing the least complicated way of managing this (having the tooth removed instead of undergoing a series of treatments) - Denial regarding tooth ache/dental infections and hope they would go away without treatment		High
Sogi, 2020 [38]		**Oral health not a priority**- 41.7% never felt the need - 27.1% lack of awareness	**Tooth brushing aids** - 75.8% used brush + paste - 12.8% used finger + powder - 4.4% used tree stick - 7% use other **Frequency of brushing** - 72.1% brush once a day - 4.8% brush teeth twice a day - 0.6% brush thrice a day - 22.4% brush occasionally **Dental visits** - 29.3% never visited dentist - 5.5% < 6 months dentist visit - 10.4% dentist visit 6–12 months - 15.2% greater than 12 months since dentist visit - 39.6% only seen dentist when there is some problem **Barriers to accessing dental care** Cost - 14.6% costly treatment Dental fear - 2.1% dental anxiety Practical barriers - 14.6% time constraint	Poor
Stevens et al., 2010 [33]	- 61% could not identify a dentist to visit		**Tooth brushing aids** - 28% of participants did not have a toothbrush/toothpaste **Frequency of brushing** - 30% of brushed twice a day - 40% brushed once a day - 25% brushed between daily and weekly - 5% less often than weekly **Dental visits** - 55% last saw a dentist over 1 year ago	High
Tani et al., 2012 [56]			**Frequency of brushing** - 26% brushed less than once a day - 29.6% brushed once a day - 18.9% brushed twice a day - 25.5% brushed 3 or more times a day	High
Teng et al., 2011 [31]	**Preventative oral health care** - 82.5% did not identify regular oral health check-ups as necessary - 50% identified dental pain as a reason to see a dentist		**Frequency of brushing** - 49% neglected tooth brushing **Dental visits** - 22.5% visited dentist within 1 year - 28% had not visited the dentist in recent years - 25% consulted dentists when they suspected an oral health problem	High
Tredget et al., 2019 [57]			**Frequency of brushing** - 50% twice daily - 31.1% daily - 9.4% every 2 days - 4.7% mouthwash only - 3.8% Never **Dental visits** - 50% less than 6 months - 23.6% only when there is problem - 18.9% Never - 3.8% annually - 3.8% every 2 years **Barriers to accessing dental care** Dental fears - 27% of patients had significant anxiety about dental care	Poor
Villadsen et al., 2017 [43]		**Impact of mental illness**- Symptomology of mental illness affected ability to take care of oral health **Oral health not a priority**- Oral health not assigned a priority among other self-care priorities		High
Waplington et al., 2000 [47]			**Barriers to accessing dental care** Cost - Two residents expressed financial reasons prevented them from attending the dentist	Poor
Wright et al., 2021 [44]		**Oral health not a priority**- Some participants considered oral health a low priority	**Barriers to accessing dental care** Cost - cost/limited income/lack of dental insurance - socioeconomic status Dental fear - dental anxiety - fear of pain - negative experiences with dental care - fear of suffocation Practical barrier - Expressed transportation as a barrier **Poor communication with dentist and health care providers** - perceived lack of empathy - lack of communication by dentists - lack of communication on oral health by psychiatrists	High
Yoshii et al., 2017 [61]			**Tooth brushing aids** Use of fluoride toothpaste Yes = 45.8%, No = 31.7%, unaware of toothpaste content 22.5% Use of interdental brushes or floss Everyday = 14.8%, sometimes 20.4%, No = 64.8% **Frequency of brushing** Tooth brushing before bed Everyday M = 32.7%, F = 27.9%, Sometimes = M 11.5%, F = 4.3%, No = M 18.3%, F = 5.3% Tooth brushing after meals Ever day M = 20.1%, F = 16.4%; Sometimes M = 28.6%, F = 16.4% No M = 13.8%, F = 4.6% Of those in the program at Baseline (*n* = 142) Tooth brushing after meals Everyday = 38.7%, sometimes = 43.7%, No = 17.6% Tooth brushing on the go Always = 20.4%, sometimes = 26.1%, No = 53.5% **Dental visits** - Yearly routine dental visit Yes: M = 15.8%, F = 16.1%, No: M = 46.7%, F = 21.4% - 19% Regular basis - 21.8%, Go early when you have a concern - 49.3%, when there is a worrying symptom - 9.9% when there are multiple symptoms	High

**Table 4:** Quality appraisal

Author (year)	Criterion												
	1	2	3	4	5	6	7	8	9	10	11	12	13
Qualitative studies													
Bjorkvik, 2021 [40]	X	X	X	X	X	X	X	✓	✓	✓			
Ho, 2018 [42]	X	X	X	X	X	X	X	✓	✓	✓			
Kulpers, 2018 [58]	✓	✓	✓	✓	✓	✓	X	✓	✓	✓			
McKibbin, 2015 [24]	✓	✓	✓	✓	✓	✓	✓	✓	✓	✓			
Persson, 2010 [22]	✓	✓	✓	✓	✓	✓	✓	✓	✓	✓			
Villadsen, 2017 [43]	✓	✓	✓	✓	✓	✓	✓	✓	✓	✓			
Wright, 2021 [44]	✓	✓	✓	✓	✓	✓	✓	✓	✓	✓			
Mishu, 2022 [39]	✓	✓	✓	✓	✓	X	X	✓	✓	✓			
Cross sectional studies													
Agarwal, 2021 [35]	✓	✓	✓	✓	✓	X	✓	X					
Alkan, 2015 [48]	✓	✓	✓	X	✓	✓	X	✓					
Anita, 2019 [49]	✓	X	✓	✓	✓	✓	X	✓					
Bertaud-Gounot, 2013 [50]	✓	✓	✓	✓	✓	✓	X	✓					
Gurbuz, 2011 [55]	✓	X	✓	✓	✓	✓	X	✓					
Hall, 2018 [45]	✓	✓	✓	X	✓	X	X	X					
Hede, 1992 [30]	✓	✓	✓	✓	X	X	X	X					
Hede 1995 [36]	✓	✓	✓	✓	✓	✓	X	✓					
Kebede, 2012 [54]	✓	✓	✓	X	✓	✓	X	✓					
Lalloo, 2013 [51]	✓	✓	✓	✓	✓	✓	✓	✓					
Lopes, 2021 [52]	✓	✓	✓	✓	✓	✓	✓	✓					
Nayak, 2020 [53]	X	X	X	X	X	X	X	X					
Ngo, 2018 [37]	✓	✓	✓	✓	✓	✓	✓	✓					
Persson, 2009 [46]	✓	✓	✓	✓	✓	✓	✓	✓					
Sogi, 2020 [38]	X	X	X	X	X	X	X	X					
Tani, 2012 [56]	✓	✓	✓	✓	✓	✓	✓	✓					
Teng, 2011 [31]	✓	✓	✓	✓	✓	✓	✓	✓					
Tredget, 2019 [57]	✓	X	X	X	X	X	X	X					
Waplington, 2000 [47]	X	✓	X	X	✓	X	✓	X					
Cohort studies													
Stevens, 2010 [33]	✓	✓	✓	✓	✓	✓	✓	✓	✓				
Yoshi, 2017 [61]	✓	✓	✓	✓	✓	✓	✓	✓	✓				
Neilson, 2011 [60]	✓	✓	✓	✓	✓	✓	✓	✓	✓				
Khokhar, 2011 [32]	✓	X	✓	X	X	✓	X	✓	X				
Randomised controlled trials													
Adams, 2018 [34]	✓	X	✓	✓	X	✓	✓	X	✓	✓	✓	✓	✓
Almomani, 2009 [28]	✓	✓	✓	✓	X	X	✓	✓	✓	✓	✓	✓	✓
Kuo, 2020 [29]	X	✓	X	X	X	X	X	X	✓	✓	✓	X	X
Case Control													
Janardhananan, 2011 [59]	✓	✓	✓	✓	✓	✓	✓	✓	✓	✓			
Kuipers, 2022 [58]	✓	✓	✓	✓	✓	✓	✓	✓	✓	✓			

The three themes arising from the review were categorised under oral health knowledge, oral health attitudes, and oral health practices.

### Theme 1: Oral health knowledge “My medication didn’t interfere with anything “

Authors of seven studies [24, 28–33] examined oral health knowledge with one [24] of these studies being qualitative and two [28, 29] using a tool a 15-item Oral Health Knowledge questionnaire [28] and 35-item questionnaire adapted and developed from Taiwan Health Promotion School [29]. Oral health knowledge was found to be related to knowledge regarding preventative oral health practices and the side effects of psychotropic medication.

#### Preventative oral health care

Most studies reported low or basic levels of oral health knowledge irrespective of diagnosis, gender, or age [28–30, 32]. The two studies where validated tools were used, found that participants scored less than 45–50% in the oral health knowledge questions [28, 29]. Participants had limited understanding regarding preventative dental visits, and the importance of regular tooth brushing and flossing, reporting that they learned of this after experiencing significant decay or tooth loss [24]. In the only study exploring preventative dental visits, 82.5% of participants were unable to identify that regular dental check-ups were necessary for maintaining good oral health [31]. In another study, two-thirds of the participants could not identify a dentist to visit [33].

#### Side effects of psychotropic medication

Authors of two studies explored the knowledge of mental illness, psychotropic medications, and association with oral health [24, 30]. The results of one study indicated that 86% of participants did not know that medication they received may contribute to the development of cavities [30]. Similarly, McKibbin, Kitchen-Andren [24] found that nearly all participants had limited knowledge regarding medication side effects and oral health impact:

*‘No. I haven’t [talked to my dentist]. There was no need to. My medication didn’t interfere with anything as far as I know’ *[24]

*‘I don’t see how it [mental health] would have any bearing on my teeth’ *[24]

### Theme 2: Oral health attitudes- “it isn’t just a matter of brushing teeth”

The attitudes of individuals with a mental illness towards oral health were reported in 13 studies, including six quantitative [29, 34–38] and seven qualitative [22, 39–44], One study [29] used a validated tool (35-item questionnaire adapted and developed from Taiwan Health Promotion School). The oral health attitudes were summarised as, impact of mental illness and oral health not a priority.

#### Impact of mental illness

In a study conducted by Ho et al. [42] people living with mental illness identified coping with their mental health condition and dealing with life stressors as a challenge in attending to preventative oral health behaviours. This was exemplified in quotes such as the following:

*‘I don’t clean my teeth often enough. I used to clean my teeth twice a day before… I got diagnosed. You know when you get depressed you just stop showering, you stop cleaning your teeth, you stop shaving’ *[42].

Authors of three studies [39, 40, 43] identified symptomology of mental illness as a barrier to attending dental visits, with symptoms of mental illness preventing participants from making an appointment, catching transport and getting to their appointment on time [40]:

*‘On days when you feel hopeless and think of ending your life, you forget to brush your teeth and to go to the dental clinic’ *[40].

*‘Yes, and people think it’s so easy … ‘just’ a matter of brushing your teeth. But it isn’t ‘just’ a matter of brushing teeth. Some mornings, it’s like I can hardly manage to drag myself out of bed and on mornings like that I just don’t have the spare energy to go out and grab a toothbrush’ *[43].

*‘I mean I can spend days when I can’t actually get out of bed never mind think about cleaning my teeth, you know that’s just not something that’s going to happen’ *[39]

The impact of mental illness symptoms was demonstrated to not only impact attendance at dental appointments but have a further impact on mental health:

*‘My mental illness causes me to have bad periods when I need hospitalization. Then I forget appointments at the dental clinic and risk falling out and losing my treatment. The dentist thinks that I do not care or that I am a difficult person, and I feel ashamed’ *[40]

#### Oral health not a priority

Prioritisation of oral health was explored by authors in 10 studies [22, 34–39, 41, 43, 44]. Participants described oral hygiene as a challenge, one that was not assigned a priority in comparison to other self-care priorities [43]. Complementary to this, in one study authors reported that 84.7% of participants perceived oral health as having little influence in their lives [35]. In another study it was found that participants did not view oral health as a priority when experiencing symptoms of mental illness [41]:

*‘Patients have many things on their mind after a first episode psychosis; dental care is not one of them’ *[41].

*‘I have a lot on my mind, and brushing my teeth is not a priority’ *[41].

In another study by Mishu et al., [39], participants perceived that oral health was not considered important from the perspectives of mental health care providers and other health professionals:

*‘I’ve heard them say it before you know ‘we’re not experts in physical health’, but you know what you, you are my consultant psychiatrist, you are my mental health nurse, you are my social worker, you are whoever, you don’t have to be an expert in the field to put in my CPA [Care Plan Approach] letter or my discharge letter or the letter to my GP [General Practitioner]-when was the last time I saw a dentist or when’s the last time I had a physical health check . . . you know, to advocate for me and that’s what we need, we need people to support us, we need people to advocate for us’*[39].

These same participants wanted to be involved in the planning and decision making of their oral health care [39].

### Theme 3: Oral health practices- “I did not go to the dentist for over 3 or 4 years…”

In 33 studies [24, 29–61] the oral health practices of people with a mental illness were assessed. Of the 33 studies, 25 studies were quantitative [28–34, 34–39, 45, 46, 48–68], seven were qualitative [24, 25, 39–42, 44], and one study was mixed methods [47]. One study [29] used a validated tool (35-item questionnaire adapted and developed from Taiwan Health Promotion School). Oral health practices included toothbrushing aids, frequency of brushing, frequency of dental visits and barriers to accessing dental care including, cost, practical barriers, poor communication with dentists and health care providers, and dental fears.

#### Tooth brushing aids

In eight studies [32–35, 38, 49, 58, 61] the type of aid used to brush teeth, including toothbrush/toothpaste were described. A wide range of toothbrush ownership rates ranging from 38.3% [34] to 98% [58] were reported. One study with the lowest rates of toothbrush aid (38.3%) did have a smaller and younger population compared to other studies [58]. Studies that reported higher rates of toothbrush ownership had formal support services in place, including being admitted to a rehabilitation unit, having an onsite-dental clinic at an inpatient facility, or a care coordinator in the community [32–34]. In one study that reported lower rates of toothbrush use [38], it was found that participants did use other means to brush their teeth including finger and powder (12.8%) and tree stick (4.4%) which could be attributed to cultural preference.

#### Frequency of brushing

In studies that assessed frequency of brushing, rates of brushing teeth twice a day ranged from 2.7% [35] to 72.1% [33]. In three studies it was reported that 10% or less of participants brushed twice a day [35, 38, 49]. Daily brushing ranged from 31.1% [57] to 72.1% [38] with the majority of authors reporting daily brushing below 40% [33, 34, 48, 52, 57]. Although authors of one study reported high rates of toothbrush ownership (98%), frequency of brushing was low with 40% of participants brushing between once a day to a few times a-week [34]. In the one study that used a tool to assess oral health practices [29], participants scored 41% or less (4.1 and 3.6/10) on questions related to correct toothbrushing practices [29].

#### Dental visits

In 14 studies [30–33, 36, 38, 46, 48, 51, 57–61] participants’ practices regarding dental visits were assessed. The majority of participants that reported attending the dentist either in the past year or either annually or greater ranged from 15.1% [48] to 29% [32]. One study involving participants who had formal mental health support reported much higher rates (69%) of yearly dental visits [46]. In five studies, between 23.6% [48] to 80.5% [57] of participants would see a dentist as a result of having a dental concern or symptom [61].

#### Barriers to accessing dental care

Authors of nine studies [24, 38, 39, 41, 42, 44–47] identified cost as a barrier to visiting the dentist. In studies that explored cost, it was suggested that 14.6% [38] to 39% [45] of participants cited cost as a barrier to visiting the dentist with limited income, lack of insurance [44], and current financial situation [24, 41, 47] as contributing factors:

*‘I did not go to the dentist for over 3 or 4 years. Because of my debts. I am in debt restructuring; I cannot really pay the dentist’ *[41]*.*

‘The money you have to pay, it’s unbelievable’ [47].

*‘I had to come up with $1000 down plus $400 a month before he (the dentist) would even schedule me…So that took a while…cause I only [receive] $577 on disability’ *[24]*.*

In one study, a participant described that they were not able to afford necessities associated with living as well as dental care [39]:

*‘Because it’s having access to quality dental care and if it’s costing you 45 quid to go*

*now and a bit of a squirt and clean 45 quid is, you know well that’s Monday, Tuesday,*

*Wednesday, Thursday’s benefits for me well what shall we not pay? Shall we not pay my*

*rent, shall we not pay my council tax; so I am not going see my kids, yeah; no, I am okay*

*with brown teeth and a bit of plaque. You know you’re asking people to make those sort of*

*choices’ *[39].

Participants in the study by McKibbin [24], reported that dental treatment expense dictated the treatment received with participants opting for the most cost-effective option:

*‘It’s a struggle to find somewhere to help you…do fillings and stuff cause it’s a couple hundred bucks you know and that’s a lot. If you have a filling…I don’t even know, like they usually don’t…like…my Medicaid, all they do is pull them, they don’t, they won’t fill them… So it’s just, you have one cavity, you have to get the whole tooth pulled if you want to be out of pain’ *[24]

#### Poor communication with dentists and health care providers

Authors of three studies [39, 42, 44] identified poor communication as a barrier that impeded participants’ oral health. Wright et al. [44] identified a lack of perceived empathy by dentists, and lack of communication by dentists as barriers. Similarly in a study conducted by Mishu et al., [39], participants reported that they felt dental professionals lacked understanding regarding mental illness.

*‘This level of education is really needed with these groups of individuals around trauma, and you know, so that they are psychologically informed and trauma informed. You know who wants to put anybody through any kind of distress, but you know so it’s a group of people that really do need to learn more about their patients.’ *[39]

Additionally, Wright et al. [44] found that communication barriers were not isolated to dentists, with participants identifying a lack of communication on oral health by psychiatrists as a barrier.

Ho et al.,[42] identified that even though consumers may have a high level of health literacy, miscommunication still occurred. Some participants identified that the language used by dental professionals made it difficult to understand their oral health:

*‘So I think with a lot of dentists, they know what they are talking about… but we don’t. We don’t understand what they’re talking about. So it’s a bit hard to understand what they’re talking about…. I mean the way they explain it. We don’t understand all these things. You know some of the terminology. If they showed a chart or something like that or they explained it a bit more in layman’s terms… it would be a lot easier… Well it’s your teeth. You want to know what is going on with your own mouth ‘cause you can’t see. So you want to be able to know what they’re seeing especially when they are poking and probing in you’ *[42]*.*

#### Dental fears and other practical barriers

Participants in seven studies [38, 39, 42, 44–46, 57] identified fear or anxiety regarding dental visits. This ranged from 2.1% [31] to 27% [38] of participants. Additionally, practical barriers such as difficulties accessing dental providers due to distance and transportation [39, 44, 45] and time constraints [38] were also identified by participants. These practical barriers were not limited to those in the community setting, with people in inpatient settings identifying a lack of access to brushing teeth in the evening for individuals in wheelchairs, and long queues due to limited sink numbers [37].

## Discussion

This is the first systematic review to synthesis the available evidence regarding oral health knowledge, attitudes, and practices of people with a mental illness. The findings from this review indicate that individuals living with a mental illness have limited oral health knowledge around good oral health practices and the impact of psychotropic medications. Oral health is not a high priority and there are lower levels of frequency of toothbrushing and preventative dental visits among this population. The identified themes in this review particularly the limited knowledge and lack of priority in oral health seems to be a common feature observed in vulnerable and at-risk population groups like those with systemic diseases [69, 70].

Tooth brushing practices appeared to be generally low irrespective of study location, diagnosis, age, or gender. Despite current recommendations of brushing twice daily to prevent oral health disease most participants only brushed once a day, and even these rates were found to be consistently low ranging from 31.1% [57] to 72.1% [38]. Brushing twice a day is highly recommended to reduce the risk of severe periodontal disease [71] and dental caries [72] as it helps prevent the build-up of bacteria [72]. The low rates of tooth brushing frequency may be influenced by the lack of oral health knowledge evident among this population. Other contributing factors identified in this study include lower priority for oral health as well as the sense of apathy experienced by individuals living with a mental illness.

Authors of studies with an emphasis on oral health knowledge found that participants were only made aware of the importance of preventative practices when they developed a problem that required contact with a dental professional [24], suggesting that increased education and knowledge may improve preventative practices such as toothbrushing. In an interventional study, improvements were found in the frequency of brushing after participants in chronic psychiatric units were provided with education-based interventions [29].

Regular dental visits were also consistently low among this population group, with one study identifying lower dental visits and lower brushing frequency compared to the general population [58]. In studies that took place in in-patient settings [30, 32] or had ongoing psychiatric clinic visits [22], dental visits were noted to appear slightly higher than for study populations in the community settings. This suggests that mental health care providers and carers could play a role in supporting the oral health of people with a mental illness, especially as they are more likely to have more frequent contact than dental practitioners. Both mental health care providers and carers could assist in making and attending dental appointments, providing prompts to attend to activities of daily living such as attending to regular toothbrushing and providing psychoeducation regarding the oral health implications of psychotropic medications and preventative oral health practices to reduce the needs of future dental treatment.

However, before any supportive strategies can be developed it is important to consider the barriers to oral health care identified. Limited access to dental services was a key barrier and could be attributed to the perceived unaffordability of dental services resulting in a reduced number of dental visits by those with a mental illness [24, 38, 41, 45]. Financial barriers to accessing dental visits are not isolated to individuals with a mental illness with countries such as Australia reporting that approximately 39% of people over the age of 15 years avoid or delay visiting a dentist due to cost [73]. It is important to note that people living with a mental illness are likely to experience higher rates of unemployment and socioeconomic disadvantage [4] therefore impacting on affordability of dental services. Increasing accessibility to dental services and increasing awareness of services available to vulnerable populations could achieve higher rates of dental visits within this population, especially for preventative services, and reduce the long-term need requirement of invasive dental treatment.

Communication was identified as a barrier by individuals with a mental illness which was not isolated to dental practitioners, however extended to communication of oral health implications by psychiatrists. This barrier may contribute to the perceived lack of oral health knowledge among people living with a mental illness experience. It was found in this review that the language used by dentists in communicating dental care needs was also perceived as a barrier. Communication as a barrier with dental practitioners is not isolated to individuals living with mental illness, and has been found to extend into other at-risk population groups [73]. In the space of eating disorders, dieticians have reported a lack of dental professionals trained in trauma informed care which can have a negative impact on the clients’ needs [74]. Another recent study involving foster carers of children in out of home care found that the poor chairside manner of dental professionals impacted negatively on the behaviour of children especially those who were traumatised by past experiences [75]. Having increased awareness of the communication needs of people living with a mental illness, including the language used by health care providers when explaining oral health care needs, should be considered, to ensure that individuals with a mental illness can have better comprehension of shared information.

Dental anxiety and dental fear were reported in almost a quarter of studies. For these people, additional support or assistance from carers may assist in reducing anxiety or fear associated with dental visits. Additionally, considering these findings health care providers could advise carers that transportation can pose an additional barrier to attending dental visits and therefore assistance with this could increase the uptake of care as well as reduce anxiety. Although there are limited studies that have explored the carers’ role in promoting oral health within this population, it does appear that external support could play a role in oral health promotion [76].

Lastly, individuals with mental illness particularly in inpatient settings may face practical barriers in maintaining oral health like wheelchair access and availability of adequate sinks for toothbrushing. Similar issues have been cited for other vulnerable groups like those with disability [73] with many recommending making adjustments to buildings for equitable access to oral health care and regularly auditing access to such facilities [74, 77]. Similar strategies could be adopted for individuals with a mental illness when developing mental health care plans, designing models of care, and in the refurbishment of mental health units.

## Limitations

The majority of the studies reviewed included multiple conditions of mental illness and thus it was difficult to differentiate the findings based on the severity of the illness. Future studies examining single conditions or diagnoses would address this gap and add to the knowledge base. We were unable to differentiate the findings between low- and high-income countries. Furthermore, all studies included in this review that examined oral health knowledge were from higher income countries. Culture and infrastructure in countries may influence the oral health of people with mental illness and therefore this requires further exploration. Lastly, this study did not include unpublished studies, or studies that were in languages other than English, therefore there is a possibility that not all evidence in this field were retrieved.

## Conclusion

This review has highlighted to health care providers, policymakers, and researchers, some of the challenges faced by people living with a mental illness that affect their oral health knowledge, attitudes, and practices as well as their carers. The findings of this review suggests that although people with a mental illness experience barriers and challenges associated with cost, symptomology of illness, and general lack of oral health knowledge, health care providers and carers, could be involved in facilitating oral health promotion, including working in partnership with people with a mental illness to improve their oral health knowledge, attitudes, and practices. Upskilling of dental professionals in trauma informed care may promote the communication between dentists and people living with a mental illness. Additionally, providing training for mental health care providers to promote preventative dental practices may further reduce oral health disparities providing an opportunity for education and oral health care reminders for people living with mental illness. Further comprehensive and well-designed studies examining the experience of people living with a mental illness, their carers, and mental health care providers are required to improve current evidence in this area.

## Relevance for clinical practice

Health professionals providing care to individuals with a mental illness should be aware of their lack of adequate knowledge, attitude, and practices towards oral health. Health care professionals could utilise opportunities of contact with people with a mental illness to provide oral health education particularly around preventative oral health practices, such as tooth brushing and the oral health implications of psychotropic medications. Additionally, mental health providers can promote the importance of regular dental visits and aid in making and attending dental appointments along with regular audits to identify practical barriers to oral health care. Mental health care providers and dental providers also need to ensure communication with people living with mental illness regarding oral health is tailored to the needs and oral health literacy levels of people with a mental illness. Carers, both paid and unpaid, may also be able to play a role in promoting oral health within this population as demonstrated in other vulnerable population groups [74]. Mental health care providers may utilise contact with carers to provide education regarding the importance of oral health care, including tooth brushing and dental visits, for the person they are caring for. Lastly, it is important to point out that these implications for clinical practice are broad and not specific to mental illness severity and settings due to the study limitations.

### Supplementary Information

Supplementary Material 1

## Data Availability

The datasets used and/or analysed during the current study are available from the corresponding author on reasonable request.
