# Supplementary material for: Oral health knowledge, attitudes, and practices of people living with mental illness: a mixed-methods systematic review
Source: BMC Public Health. 2024 Aug 21;24:2263. doi: 10.1186/s12889-024-19713-1 (PMC11337876; doi:10.1186/s12889-024-19713-1)
Supplement: Supplementary file 1 — Supplementary Material 1 [file 12889_2024_19713_MOESM1_ESM.docx]

| S6 | (Knowledge OR attitude* OR practice* OR experience* OR view* OR perception* OR strateg* OR intervention*) AND (S1 AND S2 AND S5) | 601 |
| --- | --- | --- |
| S5 | Knowledge OR attitude* OR practice OR experience* OR view* OR perception OR strateg* OR intervention* | 2,174,977 |
| S4 | (Oral health OR oral hygiene OR dental OR periodontal OR cavity OR cavities OR mouth care) AND (S1 AND S2) | Display |
| S3 | (Oral health OR oral hygiene OR dental OR periodontal OR cavity OR cavities OR mouth care) AND (S1 AND S2) | Display |
| S2 | Oral health OR oral hygiene OR dental OR periodontal OR cavity OR cavities OR mouth care | Display |
| S1 | Mental illness OR mental disorder OR mental health OR psychiatry OR psychiatric | Display |

Search Strategy Sample: CINHAL

Search Strategy Sample: PsychINFO

| **Search Terms** | **Search Options** | **Actions** |
| --- | --- | --- |
| S25 | ( (("mental illness" OR "mental disorder" OR "mental health" OR "psychiatry" OR "psychiatric") AND (S5 OR S8 OR S18)) AND (S16 AND S19) ) NOT dementia NOT epilepsy | **Limiters** - Publication Year: 1990-2022  **Expanders** - Apply equivalent subjects  **Search modes** - Boolean/Phrase |
| S24 | ( (("mental illness" OR "mental disorder" OR "mental health" OR "psychiatry" OR "psychiatric") AND (S5 OR S8 OR S18)) AND (S16 AND S19) ) NOT dementia NOT epilepsy | **Limiters** - Publication Year: 1980-2022  **Expanders** - Apply equivalent subjects  **Search modes** - Boolean/Phrase |
| S23 | ( (("mental illness" OR "mental disorder" OR "mental health" OR "psychiatry" OR "psychiatric") AND (S5 OR S8 OR S18)) AND (S16 AND S19) ) NOT dementia | **Limiters** - Publication Year: 1980-2022  **Expanders** - Apply equivalent subjects  **Search modes** - Boolean/Phrase |
| S22 | (("mental illness" OR "mental disorder" OR "mental health" OR "psychiatry" OR "psychiatric") AND (S5 OR S8 OR S18)) AND (S16 AND S19) | **Limiters** - Publication Year: 1980-2022  **Expanders** - Apply equivalent subjects  **Search modes** - Boolean/Phrase |
| S21 | (("mental illness" OR "mental disorder" OR "mental health" OR "psychiatry" OR "psychiatric") AND (S5 OR S8 OR S18)) AND (S16 AND S19) | **Expanders** - Apply equivalent subjects  **Search modes** - Boolean/Phrase |
| S20 | (("mental illness" OR "mental disorder" OR "mental health" OR "psychiatry" OR "psychiatric") AND (S5 OR S8 OR S18)) AND (S16 AND S19) | **Expanders** - Apply equivalent subjects  **Search modes** - Boolean/Phrase |
| S19 | ("mental illness" OR "mental disorder" OR "mental health" OR "psychiatry" OR "psychiatric") AND (S5 OR S8 OR S18) | **Expanders** - Apply equivalent subjects  **Search modes** - Boolean/Phrase |
| S18 | "mental illness" OR "mental disorder" OR "mental health" OR "psychiatry" OR "psychiatric" | **Expanders** - Apply equivalent subjects  **Search modes** - Boolean/Phrase |
| S17 | S9 AND S16 | **Expanders** - Apply equivalent subjects  **Search modes** - Boolean/Phrase |
| S16 | S2 OR S10 OR S11 OR S12 OR S13 OR S14 OR S15 | **Expanders** - Apply equivalent subjects  **Search modes** - Boolean/Phrase |
| S15 | (MH "Periodontal Diseases") OR (MH "Furcation Defects") OR (MH "Gingival Diseases") OR (MH "Peri-Implantitis") OR (MH "Periodontal Atrophy") OR (MH "Periodontal Cyst") OR (MH "Periodontitis") | **Expanders** - Apply equivalent subjects  **Search modes** - Boolean/Phrase |
| S14 | (MH "Toothbrushing") OR (MH "Toothbrushes") | **Expanders** - Apply equivalent subjects  **Search modes** - Boolean/Phrase |
| S13 | (MH "Dental Care") | **Expanders** - Apply equivalent subjects  **Search modes** - Boolean/Phrase |
| S12 | (MH "Mouth Care") | **Expanders** - Apply equivalent subjects  **Search modes** - Boolean/Phrase |
| S11 | (MH "Oral Hygiene") | **Expanders** - Apply equivalent subjects  **Search modes** - Boolean/Phrase |
| S10 | (MH "Oral Health") | **Expanders** - Apply equivalent subjects  **Search modes** - Boolean/Phrase |
| S9 | S1 OR S5 OR S8 | **Expanders** - Apply equivalent subjects  **Search modes** - Boolean/Phrase |
| S8 | (MH "Adjustment Disorders+") OR (MH "Mental Disorders Diagnosed in Childhood+") OR (MH "Neurotic Disorders+") OR (MH "Organic Mental Disorders") OR (MH "Personality Disorders+") OR (MH "Substance Use Disorders+") OR (MH "Psychotic Disorders+") | **Expanders** - Apply equivalent subjects  **Search modes** - Boolean/Phrase |
| S7 | (MH "Mental Disorders+") | **Expanders** - Apply equivalent subjects  **Search modes** - Boolean/Phrase |
| S6 | Mental illness | **Expanders** - Apply equivalent subjects  **Search modes** - Boolean/Phrase |
| S5 | (MH "Mental Health") | **Expanders** - Apply equivalent subjects  **Search modes** - Boolean/Phrase |
| S4 | (Oral health OR oral hygiene OR dental OR periodontal OR cavity OR cavities OR mouth care) AND (S1 AND S2) | **Expanders** - Apply equivalent subjects  **Search modes** - Boolean/Phrase |
| S3 | (Oral health OR oral hygiene OR dental OR periodontal OR cavity OR cavities OR mouth care) AND (S1 AND S2) | **Expanders** - Apply equivalent subjects  **Search modes** - Boolean/Phrase |
| S2 | Oral health OR oral hygiene OR dental OR periodontal OR cavity OR cavities OR mouth care | **Expanders** - Apply equivalent subjects  **Search modes** - Boolean/Phrase |
| S1 | mental illness OR mental disorder OR mental health OR psychiatry OR psychiatric | **Expanders** - Apply equivalent subjects  **Search modes** - Boolean/Phrase |
